# Metastatic prostate cancer presenting as a retroperitoneal mass: a case report and review of literature

**DOI:** 10.1093/jscr/rjz291

**Published:** 2019-10-10

**Authors:** Mohamad Moussa, Mohamed Abou Chakra

**Affiliations:** 1 Head of Urology Department, Zahra Hospital, University Medical Center, Beirut, Lebanon; 2 Faculty of Medical Sciences, Department of Urology, Lebanese University, Beirut, Lebanon

## Abstract

Prostate cancer (PCa) is the second most common cancer diagnosed in men globally, after lung cancer. Many patients with PCa are asymptomatic until the tumor has progressed. The prognosis of PCa mainly depends on the presence of metastatic spread. It usually metastasizes to the bone, lung, and liver. Retroperitoneum is an exceedingly rare site for metastatic PCa to occur. We describe a case of a 68-year-old male patient presented for left flank pain and lower limb edema. A retroperitoneal mass was identified on imagery. This mass was found to be due to metastatic prostate adenocarcinoma based on immunohistochemical studies. Knowledge of this atypical presentation of metastatic PCa will reduce the diagnostic delay and allow the appropriate timely treatment.

## INTRODUCTION

Prostate cancer (PCa) has the highest cancer incidence among men in western countries and is the second most common cause of cancer death after lung cancer [1]. Stage IV PCa, consisting of stage T4 (invasion of adjacent organs), N1 (regional nodal spread) or M1 (metastatic spread) disease, is a relatively rare diagnosis, accounting for ~5% of PCa diagnoses. PCa screening may be associated with reduced rates of stage IV disease [2].

In a study of 74 826 patients where metastatic PCa was identified, the most common metastatic sites of PCa were the bone (84%), distant lymph nodes (10.6%), liver (10.2%) and thorax (9.1%) [3]. Non-regional lymphatic spread and other soft-tissue metastases are unusual and may delay the treatment. We report a case of a 68-year-old man presented with left flank pain and lower limb edema without suspicious findings on digital rectal examination of the prostate, and an elevated serum prostate-specific antigen (PSA) level of >2000 ng/ml. Radiological imaging showed a large retroperitoneal mass which was confirmed to be a metastasis from PCa by biopsy. The patient was treated by gonadotropin-releasing hormone antagonist.

## CASE REPORT

A 68-year-old male, heavy smoker with poorly controlled diabetes mellitus type 2 and hypertension, presented to the emergency department with severe left flank pain for a few days. This pain was associated with moderate lower urinary tract symptoms, progressive left lower limb edema and moderate abdominal pain. Patient denies fever, chills, vomiting, diarrhea and hematuria.

His past medical history was unremarkable except for poorly controlled diabetes (last hemoglobin A1C of 13% (normal: 4% and 5.5%) and hypertension. Physical examination revealed a diffusely red, painful and grossly swollen left lower extremity, moderate left costovertebral angle tenderness. Digital rectal examination reveals a large, non-tender, firm prostate gland with no nodular lesions. The scrotal exam was normal.

Laboratory examination revealed white blood cells (WBC) count of 9000/mm^3^, C-reactive protein of 1 mg/L, random blood sugar 420 mg/dl and creatinine 3.8 mg/dl (baseline was 1.5 mg/dl). Urine analysis showed 1–2 WBC per high-power field, other blood investigations were all within their normal range. Chest X-ray was normal.

**Figure 1:**
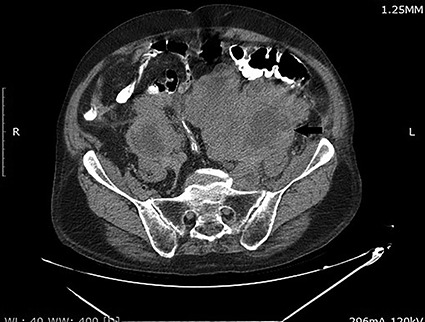
CT scan of the abdomen and pelvis demonstrating a large retroperitoneal mass (arrow).

Computed tomography (CT) scan of abdomen and pelvis with Per os contrast showed a partially necrotic and lobulated retroperitoneal mass extending along para-caval and para-aortic regions on both sides till the pelvis and enlarged prostate gland measured 60 g (Fig. 1). The left kidney shows severe hydronephrosis.

**Figure 2:**
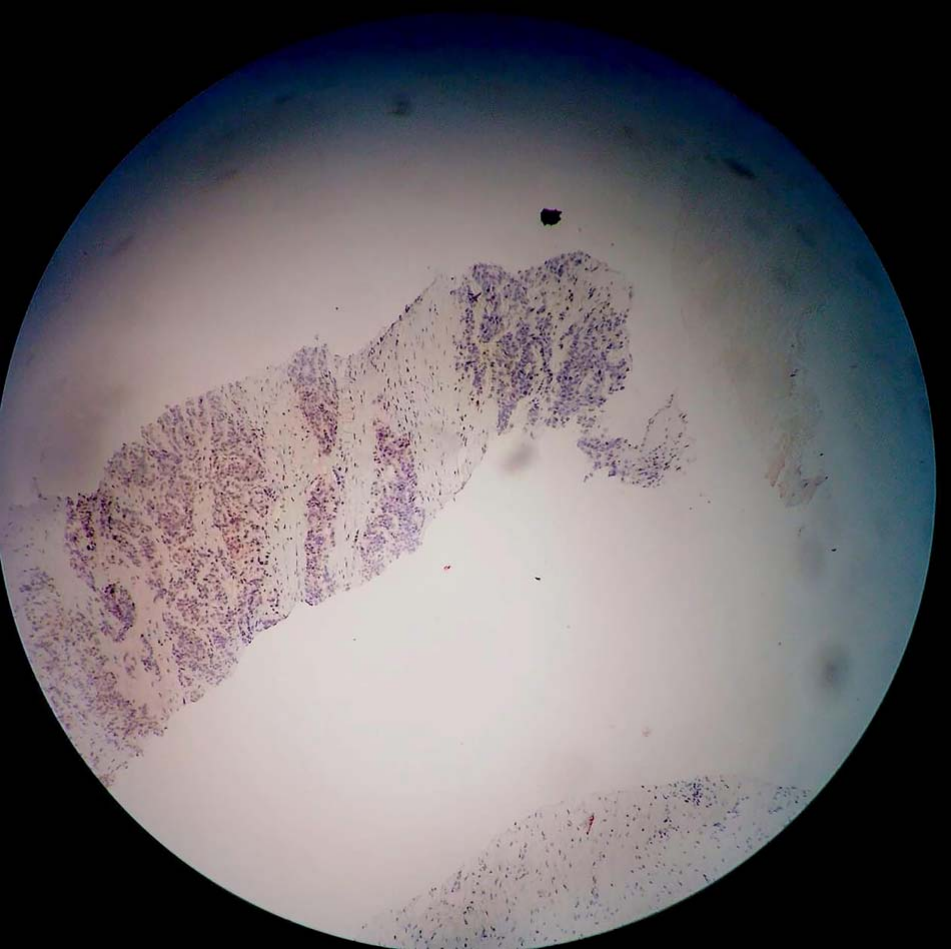
Immunohistochemical examination of the retroperitoneal mass core needle biopsy using the PSA stain.

**Figure 3:**
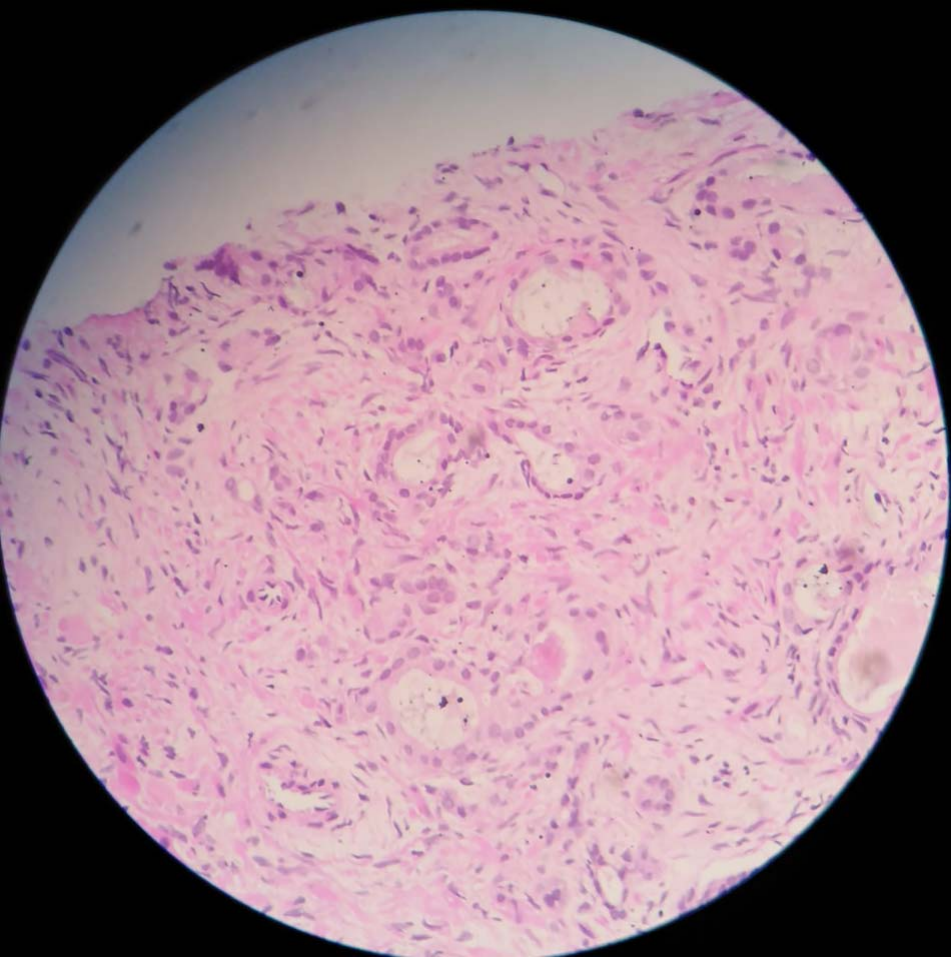
Prostate needle biopsy specimen showing prostate adenocarcinoma.

The patient was admitted to the hospital. A left double J stent was placed. PSA level was >2000 ng/ml. Core needle biopsy of the retroperitoneal mass showed thick fibrous tissue with metastatic high grade and poorly differentiated clusters of neoplastic cells consistent with carcinoma. Immunohistochemical staining reveals strongly positive results for anti-PSA (Fig. 2). These findings were consistent with metastatic adenocarcinoma of the prostate. Prostate biopsy was performed and it showed prostate adenocarcinoma, Gleason grade 8 (5 + 3) (Fig. 3).

Venous ultrasound was performed, it showed deep venous thrombosis encompassing the left distal external iliac, common femoral, femoral, popliteal and posterior tibial veins. Appropriate anticoagulation was initiated.

A bone-scan demonstrated diffusely increased uptake of sternum, lumbar spine and pelvis. Degarelix 240 mg was given initially and then 80 mg/month. The patient was offered docetaxel chemotherapy but he declined. The clinical and radiologic follow-up with abdominopelvic CT scan for 3 months shows no mass progression.

## DISCUSSION

The prognosis of PCa is essentially determined by the presence of metastases. However, the metastatic pathways in PCa are not completely understood. In an autopsy study of 1589 patients assessing the metastatic patterns of PCa, it was found that hematogenous metastases were present in 35% of the patients, with most frequent involvement being the bone, lung, liver, pleura, and adrenals. It was also found that there is a backward venous spread to the spine, which is likely to occur early in the metastatic process [4]. Long et al. [5] conducted a retrospective review of CT scans of patients with atypical PCa metastasis. The authors identified 45 atypical metastases in 36 patients. However, the sites of extranodal metastasis described were limited to the orbit and skull base (11 cases), lungs (four cases), liver (three cases), intracranial (two cases), ocular cavity (one case) and adrenal glands (one case) [5].

PCa rarely metastasizes to the Retroperitoneum. Such presentation is usually communicated as sporadic case reports. Chen et al. [6] described a 74-year-old male patient who was admitted because of dysuria and nocturia. In further evaluation, diffuse renal and retroperitoneal metastasis of PCa was confirmed. The patient was treated with radiotherapy and hormonal suppression [6]. Park et al. [7] described a 63-year-old male presented for an enlarging left supraclavicular mass and weight loss. CT scan demonstrated a large retroperitoneal mass and moderate left hydroureteronephrosis. Multiple pulmonary nodules, lytic spinal lesions, and generalized lymphadenopathy including the left supraclavicular region were seen. Biopsy of the supraclavicular node revealed prostatic adenocarcinoma [7]. Alshaikh et al. [8] presented also a case of a 67-year-old male patient with incidental findings of mediastinal and retroperitoneal masses that were found to be due to metastatic prostate adenocarcinoma based on histopathology and immunohistochemical studies. Serum PSA level was >1000 ug/L [8].

In this particular case, we learned that the atypical presentation of metastatic PCa alone would make diagnosis difficult. We reported this case to alert urologist about the rare manner, in which PCa may sometimes present. Knowledge of this atypical manifestation of metastases from PCa will reduce diagnostic delay and improve survival. PCa should be always considered in the differential diagnosis of men presenting with a retroperitoneal mass.

## INFORMED CONSENT

Written and signed informed consent was obtained from the patient.
